# Analysis of bilateral muscle coordination for characterizing neuromuscular function in postural control

**DOI:** 10.1016/j.mex.2022.101944

**Published:** 2022-11-28

**Authors:** Arunee Promsri

**Affiliations:** a Department of Physical Therapy, School of Allied Health Sciences, University of Phayao, Phayao, 56000, Thailand; b Department of Sport Science, University of Innsbruck, Innsbruck 6020, Austria; c Unit of Excellence in Neuromechanics, School of Allied Health Sciences, University of Phayao, Phayao, 56000, Thailand

**Keywords:** Neuromuscular control, Balance, Unstable surface, Stability, Homonymous muscle, Electromyography (EMG), Cross-correlation analysis, Principal component analysis (PCA), EMG, Electromyography, COP, Center of Pressure, PCA, Principal Component Analysis

## Abstract

Coordination between legs is necessary to complete bipedal balance task goals. Assessing bilateral homonymous muscle coordination may provide insight into the inherent neuromuscular control of the two legs in achieving equilibrium. This work introduced a method based on a normalized cross-correlation analysis to analyze bilateral homonymous muscle coordination during bipedal balancing on different support surfaces, revealing the temporal similarity in shape (i.e., form) between two electromyographic (EMG) signals (i.e., EMG–EMG correlation). Two levels of EMG–EMG correlation were considered: individual homonymous muscles and groups (patterns) of homonymous muscles relevant to the current task. In order to analyze the patterns of homonymous muscles, a principal component analysis (PCA) was applied to the cross-correlation coefficients to provide insights into functionally specialized groups of homonymous muscles constrained by the nervous system to work cooperatively. This proposed method has advantages that can be applied to several purposes. For example,

• Analyzing the EMG–EMG correlation provides essential information about the inherent neuromuscular function in postural control.
• At the level of individual homonymous muscles, this method can be applied to assess the neuromuscular performance after injury to the specific muscles.
• At the level of multiple homonymous muscles, this method can be used to monitor the cooperative work of several pairs of homonymous muscles in achieving equilibrium.

## Graphical abstract

**Figure:**
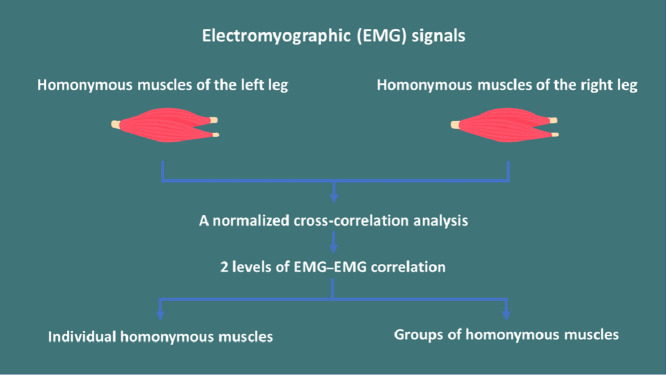

Specifications table

**Table:** 

Subject area:	Neuroscience
More specific subject area:	*Neuromuscular control* *Postural control* *Rehabilitation* *Exercise*
Method name:	*Analysis of bilateral muscle coordination*
Name and reference of original method:	*Cross-correlation analysis [**10**,**18**]*
Resource availability:	*Mathwork® (*e.g.*, xcorr and pca)*

## Method overview

### Rationale

Bipedal balance exercises have received much attention due to their effectiveness in facilitating and regaining neuromuscular control [1], e.g., gaining proprioceptive inputs [2], promoting muscle activations [3], and facilitating multiple body segment coordination [4]. Coordinating the two legs is necessary for bipedal balance activities. Bimanual coordination has been divided into high-level (cognitive or perceptual) and low-level (neuromuscular) categories, highlighting the role of the central nervous system (CNS) in the perception-action interplay [5]. At the neuromuscular level, the way the left and right limb muscles are activated into a functional control entity reflects the collaboration of the two hemispheres to carry out goal-directed motor activity [6].

Muscle coordination is commonly defined as the arrangement of the control of motor units [7] or the allocation of muscle activation among individual muscles in producing a specific joint motion [8]. Measurements of electromyographic (EMG) activity provide important information about the activities of the motor units that are needed to produce forces and generate motor behavior [9]. An overview of intermuscular coordination can be accomplished by determining the temporal similarity in shape (i.e., form) between two myoelectric signals (i.e., EMG–EMG correlation), representing the patterns of muscle coactivations (e.g., coactivation or reciprocal activations) that occur during movement generation [10]. Although performing such bilateral lower-limb motor tasks needs several effectors (i.e., muscles) to act in synchronization [11], it is still unclear whether the brain frequently or specifically utilizes the homonymous muscles of the two legs to maintain bipedal stability. The current work aimed to introduce a method to better understand the neuromuscular control of coordinated movements by focusing on how the homonymous muscles of the left and right legs cooperatively work together when performing bipedal balancing on different surfaces.

Regarding the flexibility of neuromuscular functions [12], two levels of muscle coordination—individual homonymous muscles and groups of homonymous muscles—were the focus areas. First, although individual muscles play an important and specific role according to their anatomical functions, most joints are manipulated by numerous muscles. Specifically, the same joint motion can be completed with different combinations of muscles and different levels of co-contraction or stiffness [11]. Moreover, muscle activation patterns are frequently highly individual, and not all people use the same postural movements to the same extent [4]. Second, given the numerous degrees of freedom of the motor apparatus [13], the number of variables that must be controlled can be reduced by organizing the redundancies that a specific task objective must flexibly complete into synergies (i.e., patterns) seen as both muscle synergies [14,15] and movement synergies [4,16]. In this sense, analyzing the patterns (groups) of homonymous muscles through a principal component analysis (PCA) may shed light on functionally specialized multi-muscles constrained by the nervous system to work together to achieve equilibrium [17].

In summary, this paper proposed a method to better understand the neuromuscular control of bipedal balance by focusing on the coordination between bilateral homonymous muscle activities during balancing on different support surfaces. Two levels of EMG–EMG correlation were considered: individual muscles and patterns (groups) of homonymous muscles. Knowledge of the inherent bilateral muscle coordination may benefit injury prevention and rehabilitation.

## Method details

### Method validation

Electromyographic (EMG) data were collected from 25 physically active young adults (14 males and 11 females; mean ± SD of age: 25.6 ± 4.0 yrs., weight: 71.0 ± 11.5 kg, height: 175.0 ± 8.3 cm, body mass index: 23.1 ± 2.7 kg/m^2^, and physical activity participation: 8.4 ± 5.1 h/week) when performing bipedal balancing on stable and unstable surfaces. All participants self-reported no history of neurological or musculoskeletal problems and no experience of balance-specific training for at least six months before participating in the current study. The Board for Ethical Questions in Science at the University of Innsbruck, Austria, had approved the study protocol, and the experiments were carried out under the Code of Ethics of the World Medical Association (Declaration of Helsinki). All participants signed a written informed consent before testing.

### Equipment

A Noraxon TeleMyo™ 2400T G2 Direct Transmission System (Noraxon Inc., Scottsdale, AZ, USA) with a sampling rate of 1500 Hz was used to record muscle activity. Fourteen bipolar 22-mm Ag/AgCl surface adhesive round-electrodes (Ambu Neuroline 720 01-K/12; Ambu, Ballerup, Denmark) were positioned over seven muscles of each leg; the rectus femoris, semitendinosus, biceps femoris, tibialis anterior, peroneus longus, gastrocnemius medialis, and soleus, according to the SENIAM guidelines [19]. The reference electrode was placed on the tibial tuberosity of the right leg. The electrode cables were taped to the skin to minimize movement artifacts. The low-resistance impedance between electrodes and skin (<6 kΩ) was obtained after shaving, scrubbing, and cleaning the skin. The inter-electrode distance was 20 mm. For this paper, only the tibialis anterior data are presented (Fig. 3).

**Fig. 1:**
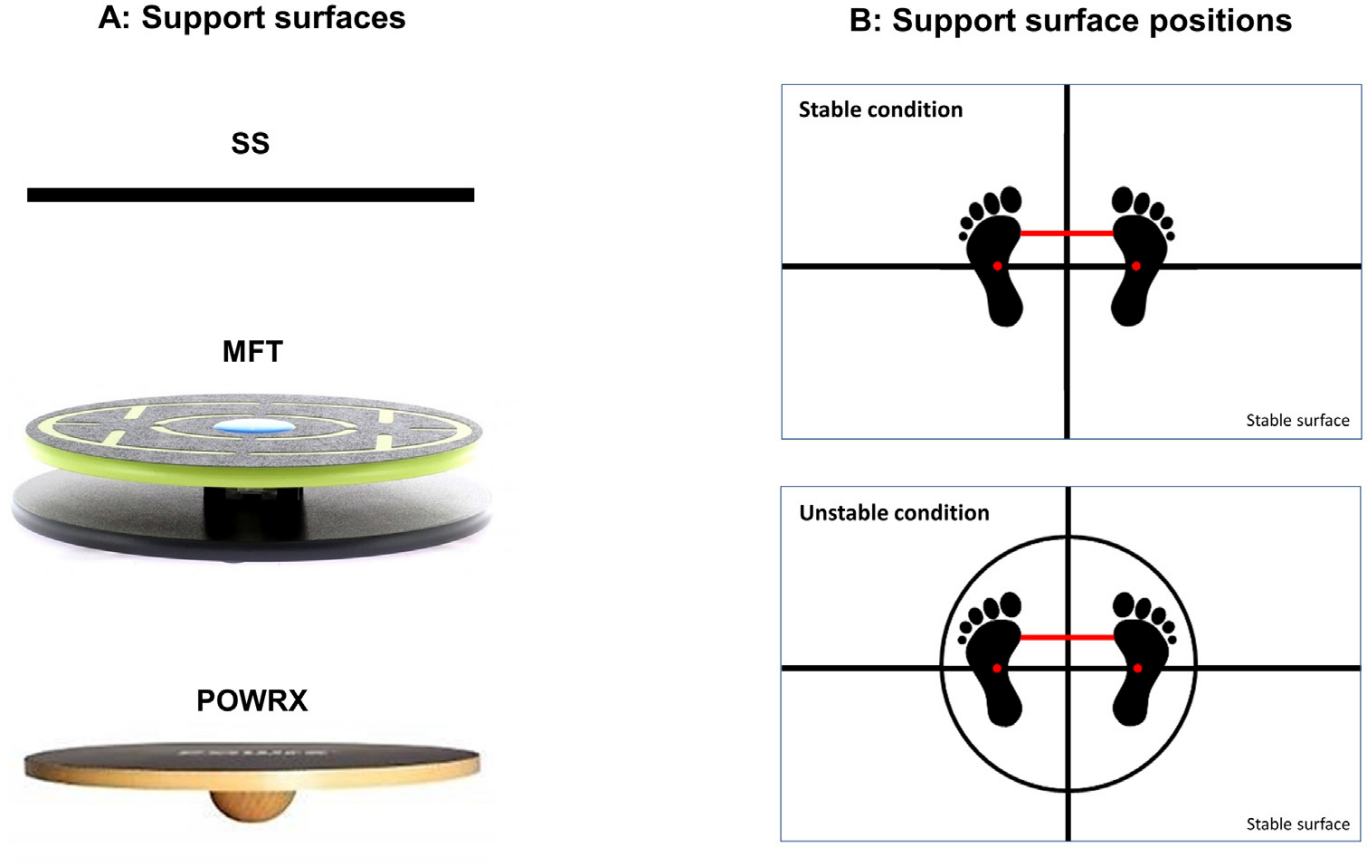
Illustrations of (A) three support surfaces: SS (stable surface), MFT challenge disk, and POWRX balance board, and (B) the standardized positions of the feet and support surfaces. Note: red dots represent the marked points on the base of the 2nd metatarsal bone of each foot. Horizontal red lines are the inter-feet distances normalized to individual bi-acromial distance. Parts of the footprints drawn in this figure were adapted from Promsri et al. [4] with permission from Elsevier.

Three support surfaces with different instability levels (i.e., difficulty levels) were selected (Fig. 1). First, a stable surface (SS) was used to induce normal-standing postural movements. Second, an MFT Challenge Disc (Trend Sport Trading GmbH., Austria) was selected to represent an intermediate difficulty, consisting of an upper 44-cm diameter round plate connected to a base circle plate by a group of four rubber cylinders with an 8-cm height at the middle of the plates. Third, a POWRX Balance Board (POWRX GmbH., Germany) was chosen to produce the greatest difficulty, consisting of a 45-cm diameter wooden plate connected with a wooden half-sphere with a 10-cm diameter and 7-cm height at the center of the board. These two balance boards allowed tilting in all directions, but the POWRX allowed for more range of motion in pivoting, rotating, tilting, or combinations of those movements.

### Experimental protocol

Before testing, each participant started with a 15-s familiarization trial on each balance board with no instruction or feedback. Then, an 80-s barefooted bipedal balancing trial on each support surface was randomly performed.

In order to standardize the starting position (Fig. 1), participants were asked to place two marked points on the base of the 2nd metatarsal bone, one on each foot, over a horizontal line taped on the floor for the SS trial or over the horizontal diameter of the balance boards for both unstable conditions, and to align the insides of the feet (the medial borders of each distal end of the first metatarsal bone) with an individual inter-feet distance (15% of bi-acromial diameter) for all trials [4].

In addition, the position of the balance boards (Fig. 1) was standardized by placing the center of each balance board over the center of a reticle crossline marked on the floor [4]. Then, marked anteroposterior and mediolateral diameters taped onto the boards were aligned to these crossed lines, in which the same fulcrum position was set for all trials of all participants.

During testing, participants were asked to look straight ahead at a target (a 10-cm diameter red circle on a white background) set at the individual's eye level on a wall approximately five meters away, stand still for stable conditions, avoid any movements not required for balancing such as scratching or rotating the head, and keep the balancing board horizontal for unstable conditions. Participants could rest (sit or stand) for one to three minutes after each trial but were not allowed to stand on the balance board.

### Data analysis: overview

The following sections introduce a normalized cross-correlation analysis used to determine the interrelationship between bilateral homonymous EMG signals. Two levels of EMG–EMG correlation: individual homonymous muscles and patterns (groups) of homonymous muscles, were the focus areas as follows:

1) Analysis of individual homonymous muscles: This step was conducted using a normalized cross-correlation analysis of each pair of homonymous muscles (i.e., EMG–EMG correlation).
2) Analysis of groups of homonymous muscles: This step was further performed using PCA on the EMG–EMG cross-correlation coefficients obtained from all the measured muscles.

**Fig. 2:**
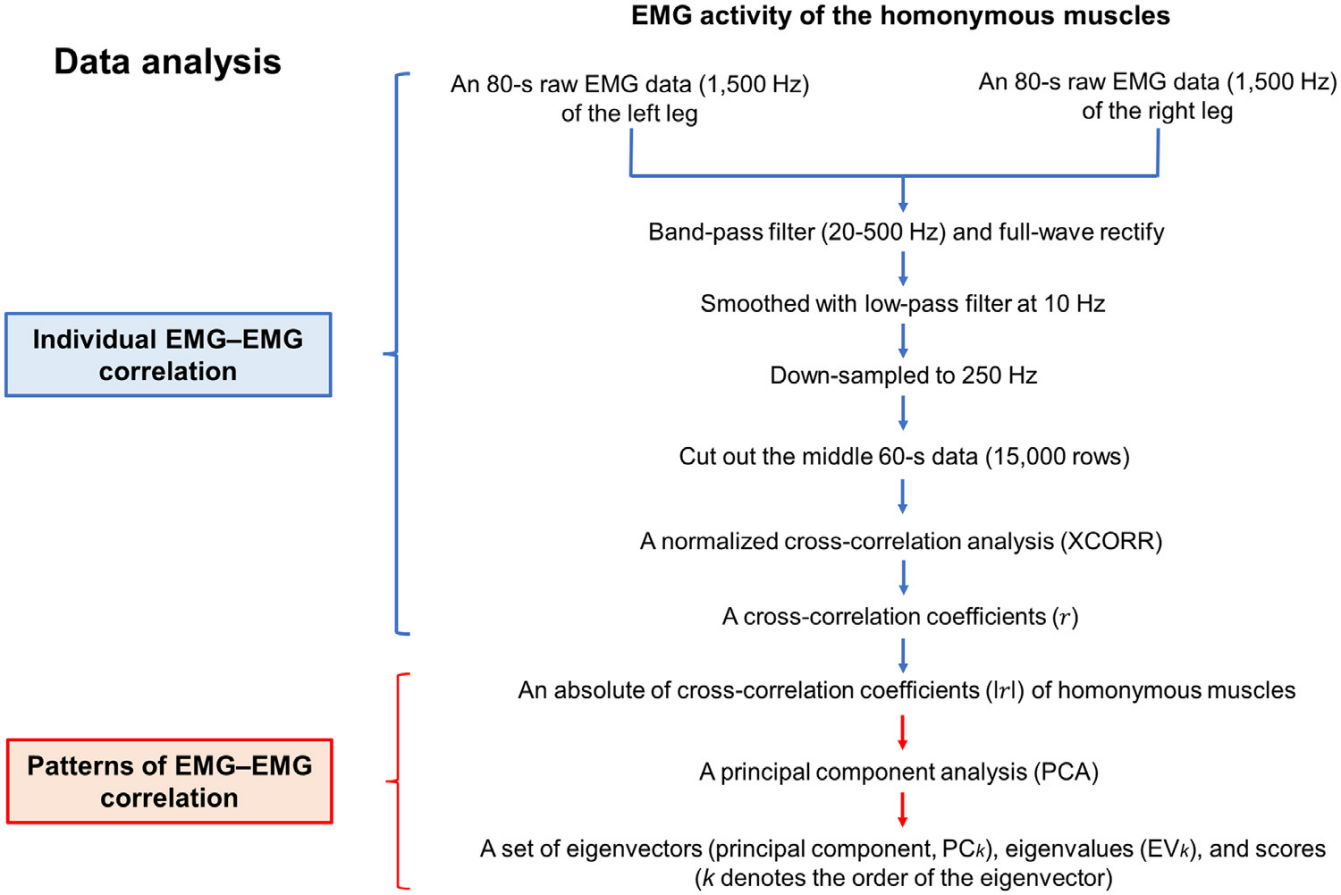
Schematic representation of data processing steps applied to the lower-leg surface electromyographic (EMG) signals.

The current methodology is introduced in the context of its prior application in a companion paper [17], which includes detailed experimental results. Fig. 2 represents a schematic representation of the data processing steps. The current study used the MatlabTM version 2020a (MathWorks Inc., Natick, MA, USA) for all data processing.

### Data analysis: individual EMG–EMG correlation

In order to prepare EMG data, individual EMG signals were filtered with a 2nd order band-pass Butterworth filter at 20–500 Hz and then rectified to remove movement artifacts and high-frequency noise [20]. Next, individual EMG signals were smoothed with the same filter, a 3rd order zero-phase 10-Hz-low-pass Butterworth filter [21]. Then, the smoothed EMG signals were down-sampled to 250 Hz, and their middle 60-s data, consisting of 15,000 timepoints, were selected for further analysis [17,21]. Individual middle 60-s EMG datasets were divided into ten subsets, each with six seconds of data (1500 timepoints) [17].

**Fig. 3:**
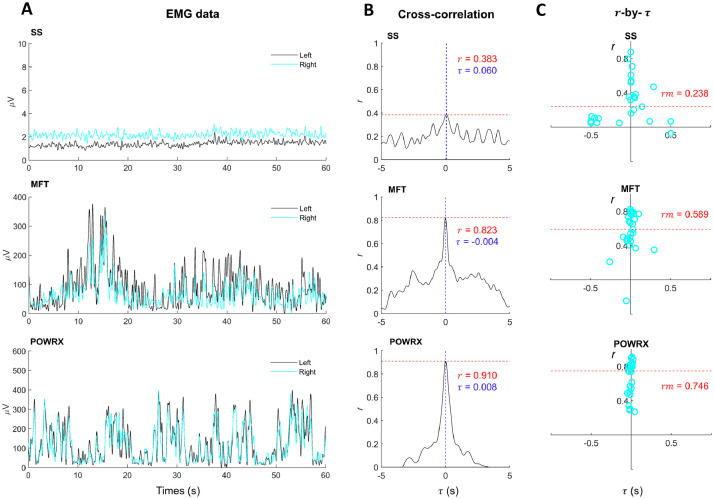
Examples of (A) the left and right leg EMG signals of the tibialis anterior muscles from one participant during bipedal balancing on all support surfaces, (B) the resultant cross-correlation graphs analyzed from the entire 60-s data, and (C) the r-by-τ graphs plotted from all participants’ data. Note: a peak (correlation coefficient; r) at the time delay (τ) observed in cross-correlation graphs indicates the level of EMG–EMG correlation. The horizontal dashed lines in the r-by-τ graphs represent the median of r (rm). The peak correlation coefficients (r) are indicated in red, while time lags at which peaks (τ) occur are indicated in blue.

A normalized cross-correlation analysis through the Matlab™ function “*XCORR*” was applied to each subset to increase the reliability of determining the cross-correlation coefficient (r)observed as a peak at the time delay (τ) in cross-correlation graphs, reflecting the shared content of these two EMG signals [18], i.e., the strength of EMG–EMG correlation. In order to interpret the strength of the correlation, the |r| value between 0.1 and 0.3 has been classified as small, between 0.3 and 0.5 as moderate, and between 0.5 and 1.0 as large correlation [22]. Fig. 3 shows the examples of (A) bilateral homonymous EMG signals from the tibialis anterior muscles, (B) the corresponding cross-correlation graphs from one participant, and (C) the r-by-τ graphs plotted from all the participants’ data. From the overview, the myoelectric activities of bilateral tibialis anterior muscles primarily increased with increased instability of the support surfaces (Fig. 3A), clearly seen as increased cross-correlation coefficients (r) (Fig. 3B). When considering all of the participants’ data, the median time delay (τm) also primarily increased with increased surface instability (Fig. 3A). Together, these findings indicated that the anterior tibialis primarily actuates in the sagittal plane, would low correlation coefficients potentially indicate an individual's tendency toward more non-sagittal plane movement/actuation. Moreover, an alignment observed at the median time delay (τm) in the r-by-τ graphs has been proposed as a criterion for determining which of the given muscles are important muscles required to maintain equilibrium [21].

For further analysis, the average value of |r| from all subsets of each volunteer was selected [10,17]. In addition, the |r| should be normalized by Fisher's r-to-z transformation if the statistical comparison is required [23].

### Data analysis: patterns of EMG–EMG correlations

In order to prepare the data for pattern analysis, the cross-correlation coefficients (r) of all subsets of each muscle were averaged and normalized through a z-score. Then, the averaged cross-correlation coefficients (r) of each participant and each balancing condition were concatenated into the same datasheet (75 rows [25 participants × 3 balance conditions] and 7 columns [7 muscles]). This step provides a comparison among three balancing conditions.

**Fig. 4:**
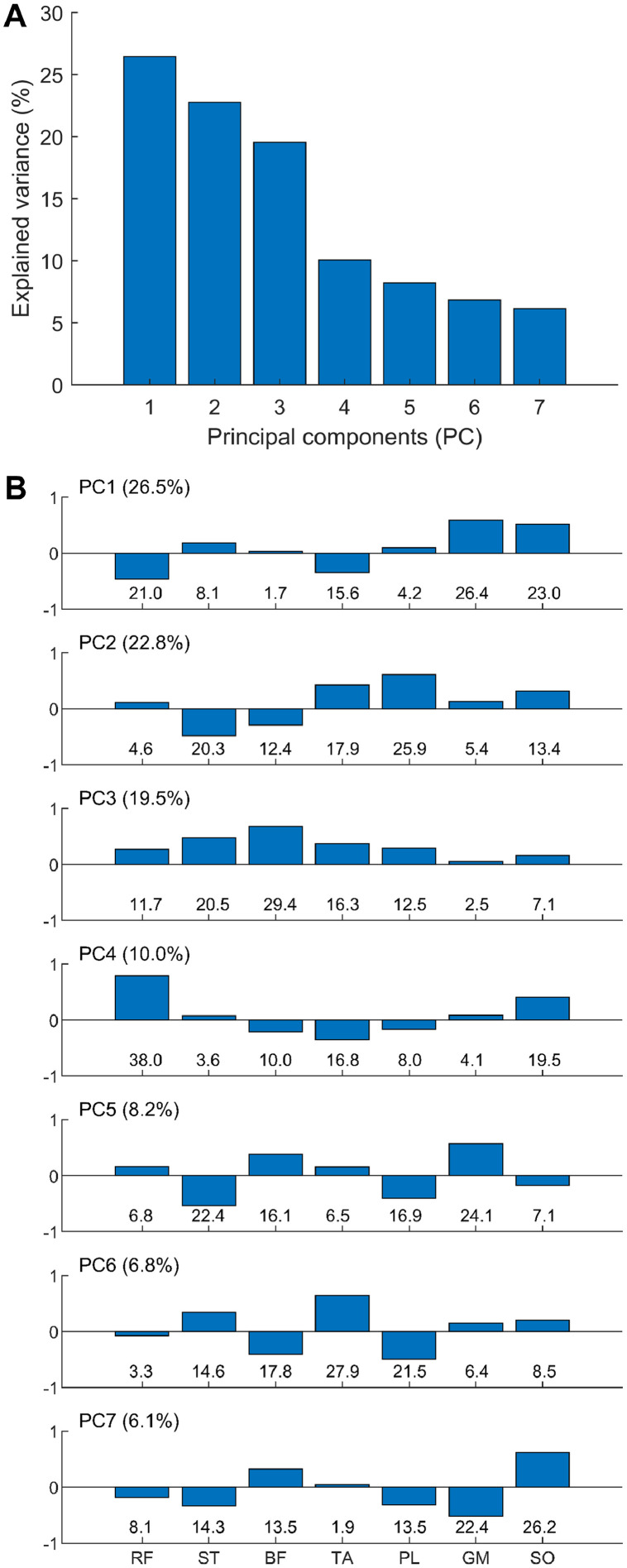
Examples of (A) the eigenvalues (explained variance in a bracket) of individual principal components (PC_1–7_) and (B) the coefficients of individual PC_1–7_ with their contributions in percent (%, bottom row) to the eigenvector.

Principal component analysis (PCA), one of the most common pattern recognition methods, through the Matlab™ function “pca” was applied to the prepared data for extracting the patterns of bilateral EMG–EMG correlations [17]. PCA provides a set of eigenvectors (principal component; PC_k_), eigenvalues (EV_k_), and scores for all trials of all participants, where _k_ denotes the order of the eigenvector. Fig. 4 shows an overview of the patterns of EMG–EMG correlations. Briefly, the eigenvalues (Fig. 4A) characterize the explained variances (%) of individual PC_1–7_ (i.e., individual bilateral EMG–EMG correlation patterns) involved in the total variances. In addition, the coefficients (Fig. 4B) represent the contribution of individual measured muscles to each EMG–EMG correlation pattern. For example, PC_1_, as the major EMG–EMG correlation pattern, explains 26.5% of the total variances, representing the positive contribution of gastrocnemius medialis and soleus muscles simultaneously acting as the main muscles seen in the first pattern.

In order to compare the patterns of EMG–EMG correlations between balancing conditions, a dependent variable, such as the relative explained variances (rVAR_k_) of the scores, can be examined as a subject-specific variable that directly corresponds to the EV_k_ to quantify how much each PC_k_ contributed to this subject's overall variance [4,24,25]. Regarding the interpretation, if differences exist in the rVAR between conditions, this indicates a difference in the patterns of EMG–EMG correlations since the participants’ overall variance influences specific PC_k_ [24].

## Limitation

The proposed method has the limitation of not considering specific muscle activation patterns, such as coactivation or reciprocal activation patterns, which were observed to have positive and negative cross-correlation, respectively [10]. However, the identification of muscle coordination strategies has been proposed as one method to detect the onset of musculoskeletal problems [26]. Therefore, considering this point may be of interest for further research.

## Conclusions

With the proposed method, an attempt was made to better understand the inherent neuromuscular control of bipedal postural balance by testing the interrelationship between bilateral homonymous EMG activities (i.e., EMG–EMG correlation) at two levels: individual homonymous muscles and groups (patterns) of homonymous muscles that are relevant to the current task goal. Analyzing the EMG–EMG correlation provides an overview of controlling bipedal postural balance, which can be applied to various clinical purposes, such as assessing neuromuscular control after ipsilateral injury or the effectiveness of a training program.

## CRediT authorship contribution statement

**Arunee Promsri:** Conceptualization, Methodology, Investigation, Software, Validation, Data curation, Visualization, Writing – original draft, Writing – review & editing.

## Data Availability

No data was used for the research described in the article.
